# Identification of nanoparticle-mediated siRNA-ASPN as a key gene target in the treatment of keloids

**DOI:** 10.3389/fbioe.2022.1025546

**Published:** 2022-10-28

**Authors:** Yipeng Dong, Chuwei Zhang, Qingrong Zhang, Zihan Li, Yixiao Wang, Jun Yan, Gujie Wu, Ling Qiu, Zhihan Zhu, Bolin Wang, Haiying Gu, Yi Zhang

**Affiliations:** ^1^ Department of Burns and Plastic Surgery, Affiliated Hospital of Nantong University, Nantong, China; ^2^ Medical School of Nantong University, Nantong, China; ^3^ Institute of Burn Research, Third Military Medical University (Army Medical University), Chongqing, China; ^4^ Institute of Analytical Chemistry for Life Science, Nantong University, Nantong, China; ^5^ School of Public Health, Nantong University, Nantong, China

**Keywords:** keloid, asporin gene, nanoparticle, keloid fibroblast, nude mice allograft, ASPN

## Abstract

**Background:** Keloid, also known as connective tissue hyperplasia, is a benign proliferative disorder with a global distribution. The available therapeutic interventions are steroid injections, surgical removal of keloids, radiotherapy, compression therapy, the application of cryosurgery, and many other methods.

**Objectives:** Existing treatments or approaches for keloids may lead to similar or even larger lesions at the site of keloid excision, leading to a high recurrence rate. Therefore, this study aims at identifying a new gene-based therapy for the treatment of keloids.

**Methods:** An ASPN-siRNA/nanoparticle combination (si-ASPN) and a negative siRNA/nanoparticle complex (NC) was developed on the basis of bioinformatics studies and used *in vitro* and *in vivo* experiments.

**Results:** The results showed a strong correlation between the development of keloids and high expression of ASPN protein. With the expression of ASPN protein greatly reduced in keloid fibroblasts and nude mice allografts after treatment with si-ASPN, the collagen and fibroblasts were also uniform, thinner, parallel and regular.

**Conclusion:** All the above experimental results suggest that keloid and ASPN are closely related and both fibroblast growth and metabolism of keloid are inhibited after silencing ASPN. Therefore, ASPN-siRNA delivered *via* nanoparticles can serve as a novel intervention therapy for the treatment of keloids.

## Introduction

Keloid, also known as connective tissue hyperplasia, is a benign proliferative disorder with a global distribution. It is characterized by the loss of normal restraint and control of collagen anabolism during skin wound healing, excessive deposition of extracellular matrix collagen and excessive growth of fibroblasts, resulting in excessive proliferation of collagen fibers invasively (Tan S et al., 2019). The lesion often interferes with normal surrounding skin tissue, exceeds the original skin damage, raising a nodular, striated or flaky mass on the skin surface with different shapes, hardness, toughness and red color. Keloids are the mostly common in people aged 10 to 30, usually located on the chest, earlobes, shoulders and back (A. Onoufriadis et al., 2018). Keloids can cause both physical and psychological burden to the patient as it may result in cosmetic impairment, itching, pain,etc. and limited joint activity in severe cases (B. Han et al., 2018). The precise etiology of keloid formation is unknown, but recent studies have pointed out a number of factors that may contribute to the keloid formation. Skin damage is one of the main causes of keloid (Finnerty CC et al., 2016; Ogawa, 2017). Clinically, a keloid forms after an inflammation or skin injury, typically months or even years after the initial event. Another significant factor related to keloid formation is genetics. There’s an increased risk of keloid after trauma of about 15%–20% in dark-skinned individuals, such as Blacks, Hispanics and Asians, which is uncommon in Caucasians, and no cases have been reported in albinos (Gauglitz, G.G. et al., 2011).

Asporin (ASPN), an extracellular matrix protein, is a member of a family of small leucine-rich proteoglycans that has been found to play an important role in collagenogenic fibril production, signal transduction and tumour growth. Recent studies have reported its role in the microenvironment of various types of cancers, including breast, pancreatic, prostate, and colorectal cancers. It has also been reported that ASPN secreted by cancer-associated fibroblasts can promote cancer cell invasion and metastasis (Simkova D.et al., 2016; Castellana B.et al., 2012; Maris P.et al., 2015; Hurley PJ.et al., 2016; Wang L.et al., 2017; Huo W.et al., 2016). Asporin has also been considered to be a beneficial regulator in cardiac remodeling (Chengqun Huang.et al., 2022). Previous studies have also shown an association between ASPN, keloid gene expression and increased protein expression (Liu L et al., 2021). Therefore, the ASPN may play an important role in the formation of keloids.

**FIGURE 1:**
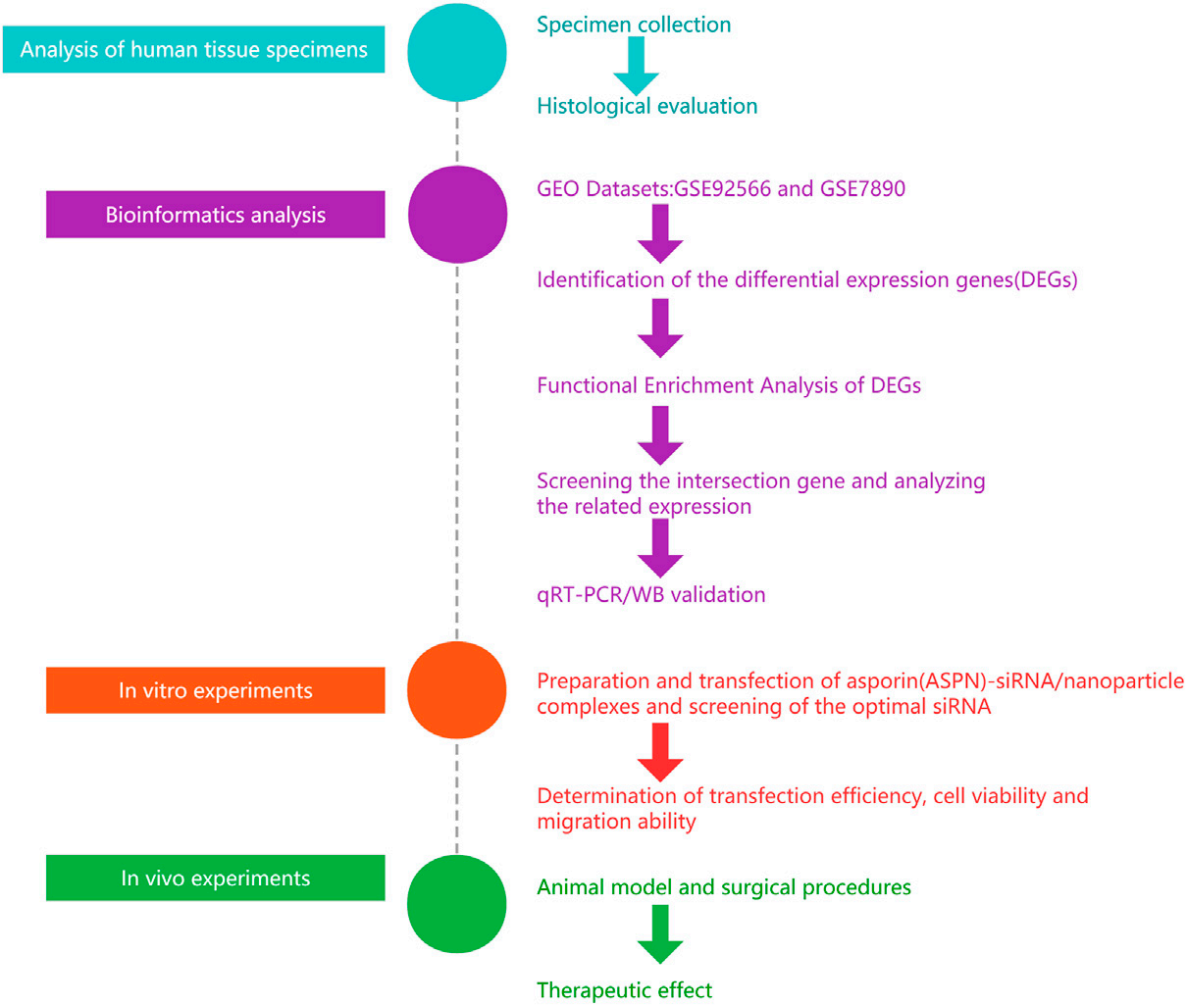
General flow chart of the experiment. We used bioinformatic analysis to first identify genes that were differentially expressed between diseased and normal skin. Then ASPN was confirmed as a crucial gene. The ideal ASPN-siRNA was then created and tested, and it was loaded onto PLGA nanoparticles for an *in vitro* investigation to evaluate its characteristics and transfection effectiveness. For *in vivo* research, we transplanted patient-provided keloid samples into nude mice. To assess the therapeutic effects after transplantation, we administered si-ASPN/nanoparticle complexes into the keloid tissue.

Particles with a diameter of roughly 1–1000 nm are known as nanoparticles (Yan-xiong Wu et al., 2017; Sun et al., 2015). When transported to the target cell, nanoparticles can protect payloads such as vaccine antigens, proteins, nucleic acids, and medicines from destruction. (Grabowska et al., 2021; Padilha et al., 2021; Yousefi et al., 2021). Compared to viral vectors, nanoparticles are non-viral delivery vehicles, easier to process and modify, biocompatible, biodegradable, and more favourable safety profiles (ShenJ et al., 2013; S. Arany et al., 2013; Y.C. Chung et al., 2010; M.E. Davis et al., 2010). The slow release properties of nanospheres facilitate sustainability of keloid treatment (DelgadoD et al., 2012; Wong SP et al., 2012). Therefore, it was hypothesized that the ASPN-siRNA/nanoparticle combination would be effective in inhibiting the growth of keloid. Hence, in this study, we applied bioinformatics analysis to first identify genes differentially expressed between diseased and normal skin tissues and ASPN was identified as a key gene. The ideal ASPN-siRNA was developed, tested, and loaded onto PLGA nanoparticles for an *in vitro* investigation to evaluate its characteristics and transfection efficacy. For *in vivo* trials, we transplanted patient-based keloid samples into nude mice to assess the therapeutic impact after transplantation, we administered si-ASPN/nanoparticle complexes into the keloid tissue (Figure 1).

## Material and methods

### Tissue samples

A retrospective analysis of the case data accumulated during our clinical work was performed to guide the study. Human keloid tissue was obtained from 10 keloid patients who underwent surgery at Affiliated Hospital of Nantong University (Jiangsu province, China) from January 2022 to May 2022, while 10 cases were obtained from normal skin tissue of other plastic surgery patients.

Inclusion criteria: ①Selection of patients with pathologically confirmed or diagnosed with keloids to obtain pathological tissue samples; ② Selection of patients who choose other plastic surgery procedures to obtain normal skin tissue; ③ Patients with complete clinical information and signed informed consent were included. Exclusion criteria: ① Patients who have been treated for keloid in the past; ②Patients with fibroproliferative diseases, such as Dupuytren disease; ③ Patients with skin infections and subcutaneous nodules in the area of the keloid; ④ Patients diagnosed with autoimmune diseases, malignancies, haematological diseases, serious infectious diseases, serious respiratory diseases and circulatory diseases or severe kidney and liver dysfunction.

The Affiliated Hospital of Nantong University’s Ethics Committee gave its approval to the current study. All participants affirmed their approval to the publication of the photos by signing an informed consent form and a statement. A pathologist with experience in the area analyzed each specimen for keloid growth. (NO.2022-K142-01)

### Download and preprocessing of microarray data for bioinformatics analysis

The NCBI-Gene Expression Omnibus (GEO) database, a global public gene data repository run by the National Center for Biotechnology Information (NCBI) of the National Library of Medicine, provided the source data for this study (https://www.ncbi.nlm.nih.gov/geo/). The GEO data information includes genes, proteomic analyses, non-coding RNA analysis, etc. The majority of worldwide research organizations maintain these data with their original study findings. For this study, the GEO database’s two keloid-related datasets, GSE92566 and GSE7890 mRNA expression profiles (Homo sapiens), were retrieved. The authors of GSE92566 are Fuentes-Duculan J et al. It was created using the [HG-U133 Plus 2] Affymetrix Human Genome U133Plus2.0 array on the GPL570 platform. For this experiment, six samples from GSE92566 were selected, including two groups (three keloid lesions and three adjacent non-lesioned samples; in addition, we excluded one newly formed keloid sample from GSE92566 for better data consistency). The GSE7890 microarray dataset [(HG-U133 Plus 2) Affymetrix Human Genome U133 + 2.0 array], which contains 10 samples, was also annotated on the GPL570 platform (five keloid, five normal skin).

### Identification of differentially expressed genes

The platform files and sequence matrices were downloaded, and R (V4.1.2) (https://www.bi-oconductor.org/packages/release/bioc/html/limma.html) was used to convert the gene IDs, merge datasets, analyze potential batch effects, normalize data, and calculate gene expression. The probes averaged as the final expression of the genes matched by multiple probes were downloaded. The Limma package was then used to identify probes that were differentially expressed in keloid compared to normal tissue samples. Adjusted p-values were accessed after performing multiple testing corrections using the Benjamini-Hochberg (BH) method. To distinguish between up-regulated and down-regulated genes, absolute log2 fold increases larger than 1.5 and adjusted p-values less than 0.05 were used as thresholds.We selected the differentially expressed genes (DEGs) depicted on the volcano plot. Using the ggplot2 package deg, prominent DEGs were simultaneously plotted as a heat map in R (V4.1.2) (Gu et al., 2016).

### Functional enrichment analysis of differentially expressed genes

R package clusterProfiler (V3.14.3) and ggplot2 were used to analyze the DEGs for gene ontology (GO) functional enrichment and Kyoto Encyclopedia of Genes and Genomes (KEGG) analysis to better understand the biological activities of genes (V3.3.3) (Yu et al., 2012). Further Gene Set Enrichment Analysis (GSEA) (Subramanian A et al., 2005) was performed to determine the underlying molecular mechanisms or potential functional pathways for comparing keloid and skin tissue based on a *p* value <0.05.

### Screening the intersection gene and analyzing the related expression

Lasso Cox regression and Support Vector Machine (SVM) dimensionality reduction algorithm were used to screen for significant genes among the DEGs, and “glmnet” package (V2.1.1) and “kernlab” package (0.9–31) were used respectively. Simultaneously, the “Venn"packages were used to screen the intersection genes and Venn diagram was drawn, in which the overlapping part of the two circles represented the intersecting genes. Boxplots were generated using the “ggpubr"package to analyze the expression of each intersection gene in keloid (test) group and normal (control) group. The ROC curve was plotted using the R package “pROC” (v1.8) to analyze the sensitivity and specificity of each related gene (Robin X et al., 2011).

### Quantitative real-time PCR and western blot analysis

Total RNA was extracted from normal and keloid samples using Invitrogen TRIzol reagent according to the manufacturer’s instructions (ThermoFisher Scientific, China). Next, total RNA was reverse cleaved using the Human-ASPN test kit according to the manufacturer’s protocol (Shanghai Rui Mian Biological Technology Co. Ltd.) into cDNA followed by qPCR. Relative expression levels were normalized to the final control GAPDH. (Human-ASPN-F Seuence (5’→3′): TGG​GAG​TCT​TGC​TAA​CAT​ACC; Human-ASPN-R Seuence (5’→3′): CAT​CTT​TGG​CAC​TGT​TGG​AC; Human-GAPDH-F Seuence (5’→3′): CTG​GGC​TAC​ACT​GAG​CAC​C; Human-GAPDH-R Seuence (5’→3′): AAG​TGG​TCG​TTG​AGG​GCA​ATG). To assess the total protein content, tissues or cells were treated with protein lysate (Servicebio, Wuhan, China) containing Phenylmethanesulfonyl fluoride (PMSF) followed by Western blotting analysis.Antibodies used included: rabbit anti-ASPN antibody (DF13642,Affinity, China), rabbit anti-GAPDH antibody (Huabio, Hangzhou, China), Anti-rabbit IgG (H + L) (Cell Signaling Technology,United States).The results of relative quantification were analyzed using ImageJ greyscale scanning software.

### Histological evaluation and immunohistochemical staining analysis

The acquired sample segments were submerged in 10% paraformaldehyde for 24 h. A paraffin microtome (RM2235, Leica, Wetazlar, GER) was used to slice samples embedded in paraffin blocks into 5-m thick sections.The slides were rehydrated dried and given a PBS washing. The slides were subsequently stained with hematoxylin and eosin (HE) (Servicebio, Wuhan, China) for histological analysis to gauge the percentage of inflammatory cells. Masson’s trichrome staining (Servicebio) was used to examine the density of collagen fibers and variations in collagen under a microscope.Subsequently, the slides were then treated with rabbit anti-ASPN antibody, rabbit anti-SMA antibody, and rabbit anti-collagen I antibody overnight at 4°C each and incubated with the secondary antibody at room temperature for 2 h. The slides were next stained with Meyer’s hematoxylin and 3,3-diaminobenzidine (DAB; Sigma, St. Louis, Missouri, United States) for 1–2 min (Sorabio, Beijing, P.R.C.). After blocking with neutral gum (Invitrogen, San Diego, CA, United States), the slides were examined under a microscope (Leica DMR 3000; Leica, Bensheim, Germany). ImageJ was used to quantitatively analyse the immunohistochemistry image data.

### Preparation and transfection of si-ASPN/nanoparticle complexes

Three double-stranded siRNAs for *in vitro* testing in accordance with the genetic sequences of Human ASPN to identify the gene sequence of a siRNA that greatly reduced the production of the target gene. The sequences (5′to 3′) of the three ASPN (human)siRNA are ASPN (human) siRNA1:/rG//rG//rA//rG//rU//rA//rU//rG//rU//rG//rC//rU//rC//rC//rU//rA//rU//rU//rA/TT,/rU//rA//rA//rU//rA//rG//rG//rA//rG//rC//rA//rC//rA//rU//rA//rC//rU//rC//rC/TT; ASPN (human) siRNA2:/rG//rC//rC//rA//rU//rU//rU//rU//rU//rU//rU//rC//rC//rA//rU//rU//rU//rG//rA/TT,/rU//rC//rA//rA//rA//rU//rG//rG//rA//rA//rA//rA//rA//rA//rA//rU//rG//rG//rC/TT, and ASPN (human) siRNA3:/rC//rC//rU//rU//rU//rC//rU//rA//rA//rC//rC//rA//rC//rA//rA//rA//rG//rA//rA/TT,/rU//rU//rC//rU//rU//rU//rG//rU//rG//rG//rU//rU//rA//rG//rA//rA//rA//rG//rG/TT. Both strands of each siRNA, containing the negative control (negative siRNA), were commercially synthesized (GenePharma, Shanghai, P.R.C.) and then annealed to form a double-stranded oligonucleotides. The actual molecular weight differs from the theoretical molecular weight by less than 0.05%. Nanoparticles have been reported to be obtained using a double emulsion method (Yang et al., 2018; Zhou YL et al., 2019). The main component of the nanoparticles is polyis poly (D,L-lactide-co-glycolide) [PLGA, lactide:glycolide (65:35), Mw = 40–75 kDa] from Sigma-Aldrich (St. Louis, MO, United States). The nanoparticles were modified with polydisperse branched polyethyleneimine (PEI, Mw = ∼25 kDa, Sigma, St. Louis, Mo., United States) and loaded with siRNA to attract negatively charged siRNA with a positive charge. The final concentration of nanoparticles obtained was approximately 1 μg/μl. To develop ASPN siRNA/nanoparticles (si-ASPN) and negative siRNA/nanoparticles (NC), the nanoparticle solution was combined with PEI in deionized water. The combination was then gently centrifuged at a N/P ratio of 6:1 (polyethyleneimine molar to DNA phosphate molar) (Figure 5A).

### Culture of primary keloid fibroblasts

Previous studies have demonstrated that keloid formation could be mainly caused by abnormal keloid fibroblasts associated with the microenvironments of keloid lesions (Xianglin Dong ei al., 2013). Our pathological keloid tissue was taken from keloid patients with complete excision of subcutaneous tissue. The samples were then sliced into 5 mm × 5 mm size and digested with 0.25% Dispase II (D6430, Solrabio, China) for 4 h to completely separate the epidermis from the dermis. The epidermal tissue was removed and dermal tissue retained. The retained dermal tissue were minced and digested with 1 mg/ml collagenase type I (C917425, Macklin, China) for 3 h, followed by filtration through 50-µm strainers to obtain fibroblasts. The keloid fibroblasts were subcultured in 10% FBS in DMEM supplemented with 1% penicillin and streptomycin. Culture dishes were placed in a moist atmosphere of 5% CO_2_ at 37°C.All primary fibroblasts were used before reaching the fifith passage.

### Screening of the optimal siRNA

The 24-well plates were filled with keloid fibroblasts until each well’s cell density reached 70%–80%. Three different types of ASPN siRNA/nanoparticles were diluted with 1 mL of culture medium (1 ml of each contained 2.5 µl of nanoparticles and 2.5 µl of ASPN siRNA). Keloid fibroblasts in well plates were divided into four groups. Keloid fibroblasts that had not been interfered with served as the control group, whereas keloid fibroblasts that had been transfected with one of three different ASPN siRNA/nanoparticle complexes—siRNA1, siRNA2, or siRNA3—served as the experimental groups. They underwent a 24-h CO_2_ incubation at 37°C and Western blot was used to detect ASPN protein expression.

### *In vitro* transfection efficiency

To investigate the transfection effectiveness of the of the ASPN siRNA/nanoparticle (si-ASPN) complex in keloid fibroblasts *in vitro*. Cells were seeded in 24-well plates until roughly 70%–80% coverage was reached in each well. Using a fluorescent microscope (Leica DMR 3000; Leica Microsystem, Bensheim, GER) equipped with GFP channels, fibroblasts were grown in non-transfected (Control) or ASPN siRNA/nanoparticle (si-ASPN) mixture for 24 h (excitation 488 nm, emission 507 nm). Then, the cells were collected in plates, suspended in PBS, and a FACSCalibur flow cytometer was used to assess the transfection efficiency (BD FACSCalibur, BD Bioscience, San Jose, CA, United States).

### Cell viability and cell migration ability

The EdU (5-ethynyl-2′-deoxyuridine) test was used to detect the keloid fibroblast growth. The total number of cells and the number of EdU-positive cells were counted under a microscope. Positive cells are calculated by dividing the total number of positive cells by the percentage of all cells using ImageJ. To assess keloid fibroblasts’ capacity for cell migration, a scratch test was employed. The state of the wound was assessed every 24 h, and photographs are obtained under a light microscope at 48 h. ImageJ was used to calculate wound healing rate = (0 h scratch area—48 h scratch area)/0 h scratch area x 100%.

### Animal model and surgical procedures

In accordance with The Affiliated Hospital of Nantong University’s Guidelines for Care and Use of Laboratory Animals, and the Nantong University Animal Ethics Committee Approval to all animal treatment operations (Animal ethics: S20220515-902), animal models for this study included six-week-old Foxn1nu BALB/c male nude mice (Laboratory Animal Center of Nantong University, Nantong, China). Six nude mice were randomly divided into two groups of three each. They were a part of the negative control group (NC) group treated with negative siRNA/nanoparticles and the si-ASPN group treated with ASPN-siRNA/nanoparticles, respectively. In a sterile setting, the diseased keloid tissue was divided into twelve pieces, each weighing roughly 105.2 ± 6.6 mg. A 5-mm transverse incision was made, and a pair of segments were put into the incisions on each side of the nude mice’s spine and the incisions were stitched up with non-absorbable sutures. These nude mice were raised for 7 days for the keloid tissue samples could integrate into the animals’ subcutaneous tissue, and they were freely fed and drink throughout a typical light/dark cycle. After 7 days, keloid tissues including the si-ASPN group were injected separately with a 100 µl mixture containing 24 µl ASPN siRNA/nanoparticles (containing 12 µl nanoparticles and 12 µl ASPN siRNA) and 76 µl normal saline. Keloid tissues in the NC group were injected separately with a 100 µl combination containing 24 µl negative siRNA/nanoparticles (12-L nanoparticles and 12-L negative siRNA), and 76 µl normal saline. The mice were put to death 2 weeks following the injection, and the keloid grafts were collected for examination (Figure 7A).

### Statistical analysis

The results are presented as the mean ± standard deviation (M±SD). The threshold for statistical significance was P 0.05. The Student’s t-test was used to analyze differences in protein volume, EdU-positive cells, wound closure, and graft quality. *p*-value < 0.01 in statistical analysis was considered to be statistically significant by definition. Version 8.0.2 of GraphPad Prism was used to visualize the data (GraphPad Software, San Diego, CA, United States).

## Results

### Characterization of keloid

**FIGURE 2:**
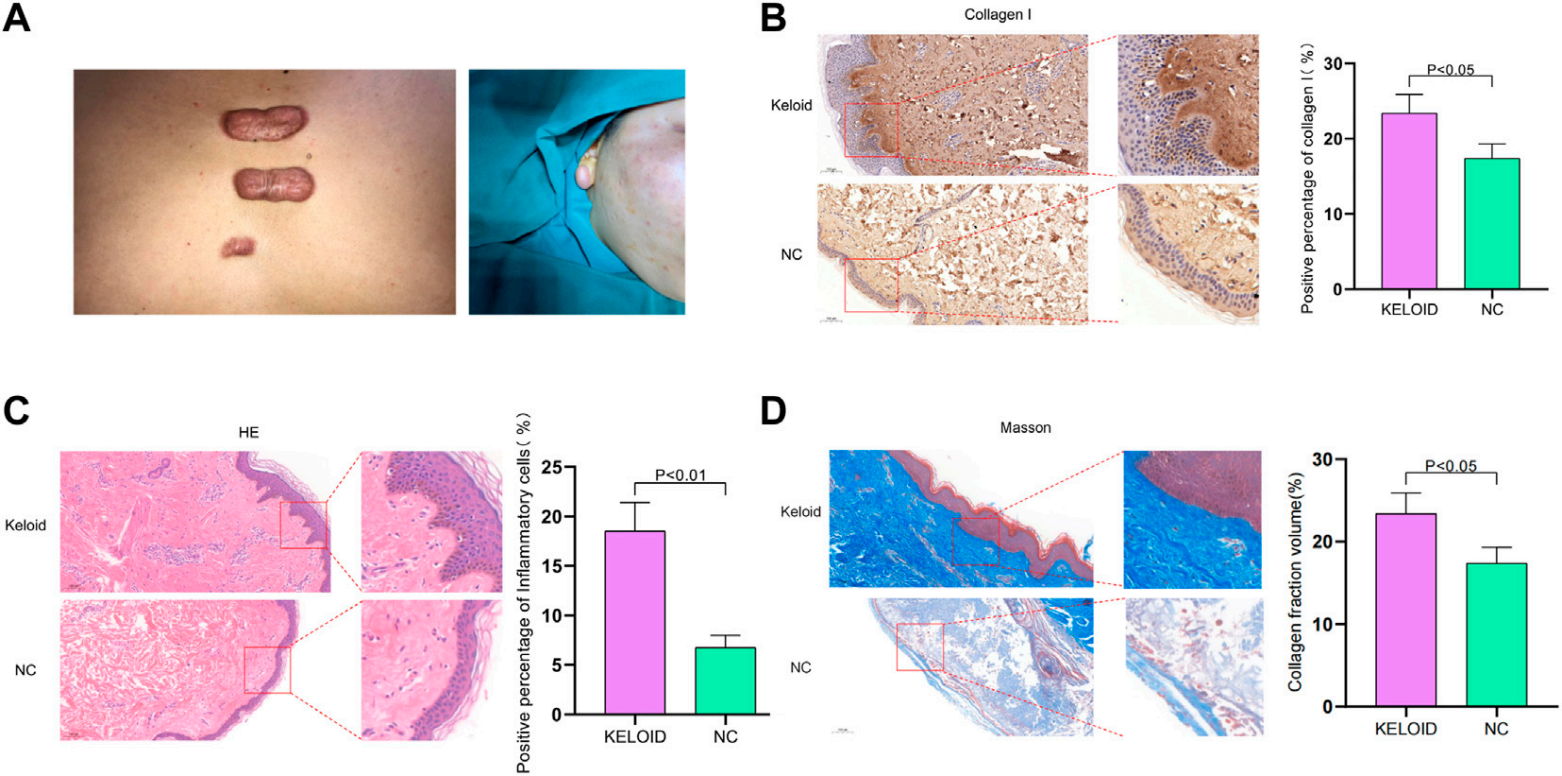
**(A)** Illustration of keloid. The keloid lesion exceeds the extent of the original skin damage and has a raised, tough surface with a reddish color. **(B)** According to histological evaluation, the keloid tissue was beyond the original wound’s margin, and had a claw-like leading edge, which is an invasive edge. The positive percentage of collagen I in keloid was clearly increased compared to normal skin tissue and the keloid tissue was clearly invading inwards. **(C)** The positive percentage of inflammatory cells in keloid was significantly higher than in normal skin tissue and the cells were dense and irregularly arranged compared to the normal skin tissue. **(D)** The collagen fraction volume was higher than in normal skin tissue. The collagen were large and thick and wavy, with an abnormal and unstable shape in keloid. Normal skin tissue has a limited margin, slender and parallel fibrocytes with regular, thin and uniform linear arrangement of collagen fibers.

As shown in Figure 2A, the keloid lesion exceeds the extent of the original skin damage and has a raised, tough surface with a reddish coloration (Figure 2A). According to histological evaluation, the keloid tissue was beyond the boundary of the original wound, and had a claw-like leading edge, which is an invasive edge. The positive percentage of collagen I in keloid was clearly increased compared to normal skin tissue and the keloid tissue was clearly invading inwards (Figure 2B). The positive percentage of inflammatory cells in keloid was significantly higher than in normal skin tissue and the cells were dense and irregularly arranged compared to the normal skin **(** Figure 2C **)**. The collagen fraction volume was also higher than in normal skin tissue. The collagen fibers were large and thick and wavy, with an abnormal and unstable shape in keloid. Normal skin tissue has a limited margin, slender and parallel fibrocytes with regular, thin and uniform linear arrangement of collagen fibers **(** Figure 2D **)**. Therefore, compared with normal skin tissue, keloid has unique characteristics.

### Differentially expressed genes in keloid

**FIGURE 3:**
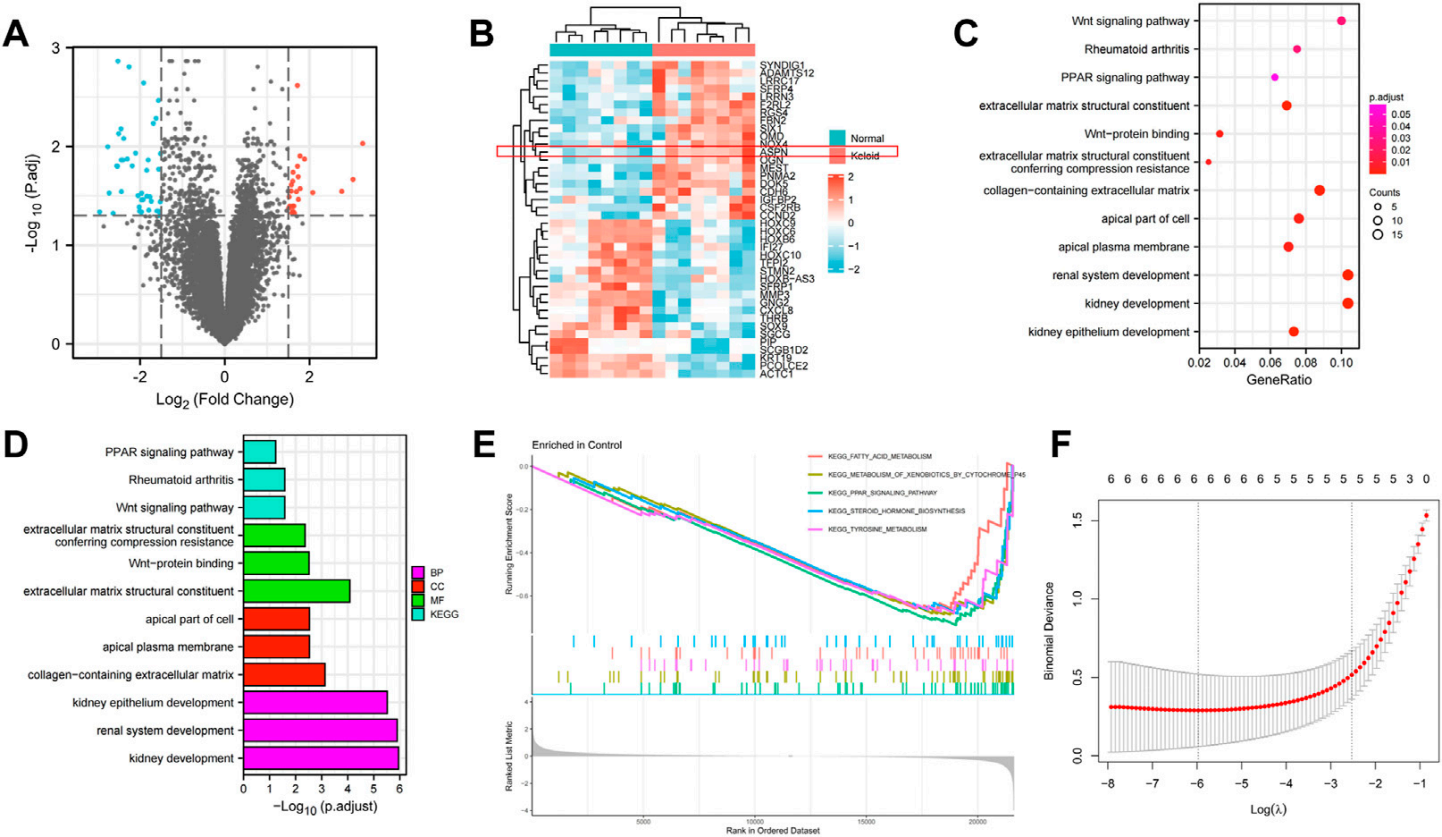
**(A,B)** A volcano map and a star cluster heat map shows the 64 DEGs between keloid and normal skin through bioinformatic analysis, including 24 up-regulated and 40 down-regulated genes. **(C–E)** Functional enrichment analysis was performed to elucidate their functions and related pathways in keloidogenesis and development, which related to biological processes like “kidney development,” “Wnt protein binding,” and “extracellular matrix structural constituent”. **(F)** The lasso Cox regression.

Sixty-four (64) DEGs were screened between keloid and normal skin through bioinformatics analysis, including 24 up-regulated and 40 down-regulated genes. The results are shown as a volcano map and a star cluster heat map **(** Figures 3A,B). Functional enrichment analysis was performed to elucidate their functions and related pathways in keloidogenesis and development. Cellular component (CC), molecular function (MF), and biological process (BP) context was supplied in the GO word analysis of DEGs. It was discovered that the above genes were related to biological processes like “kidney development,” “renal system development,” and “kidney epithelium development” in the BP entry. The DEGs for “extracellular matrix structural constituent giving compression resistance,” “Wnt protein binding,” and “extracellular matrix structural constituent” for GO MF were considerably enriched. The “apical plasma membrane,” “apical portion of the cell,” and “collagen-containing extracellular matrix” were all high in GO CC **(** Figures 3C,D **)**. Wnt signaling pathway, rheumatoid arthritis, and PPAR signaling pathway are the other three ways included in KEGG. A gene set enrichment analysis (GSEA) was further performed to elucidate the key pathways involved in the DEGs **(** Figure 3E **)**. Samples classified as ‘fatty acid metabolism','metabolism of exogenous substances by cytochrome P450’, ‘PPAR signaling pathway','steroid hormone biosynthesis’ and ‘tyrosine metabolism’were the closest in the degree of enrichment. Therefore, it was hypothesized that these genes may affect the development of keloid by participating in the above biological processes and pathways.

### Screening the intersection gene and analyzing the related expression

**FIGURE 4:**
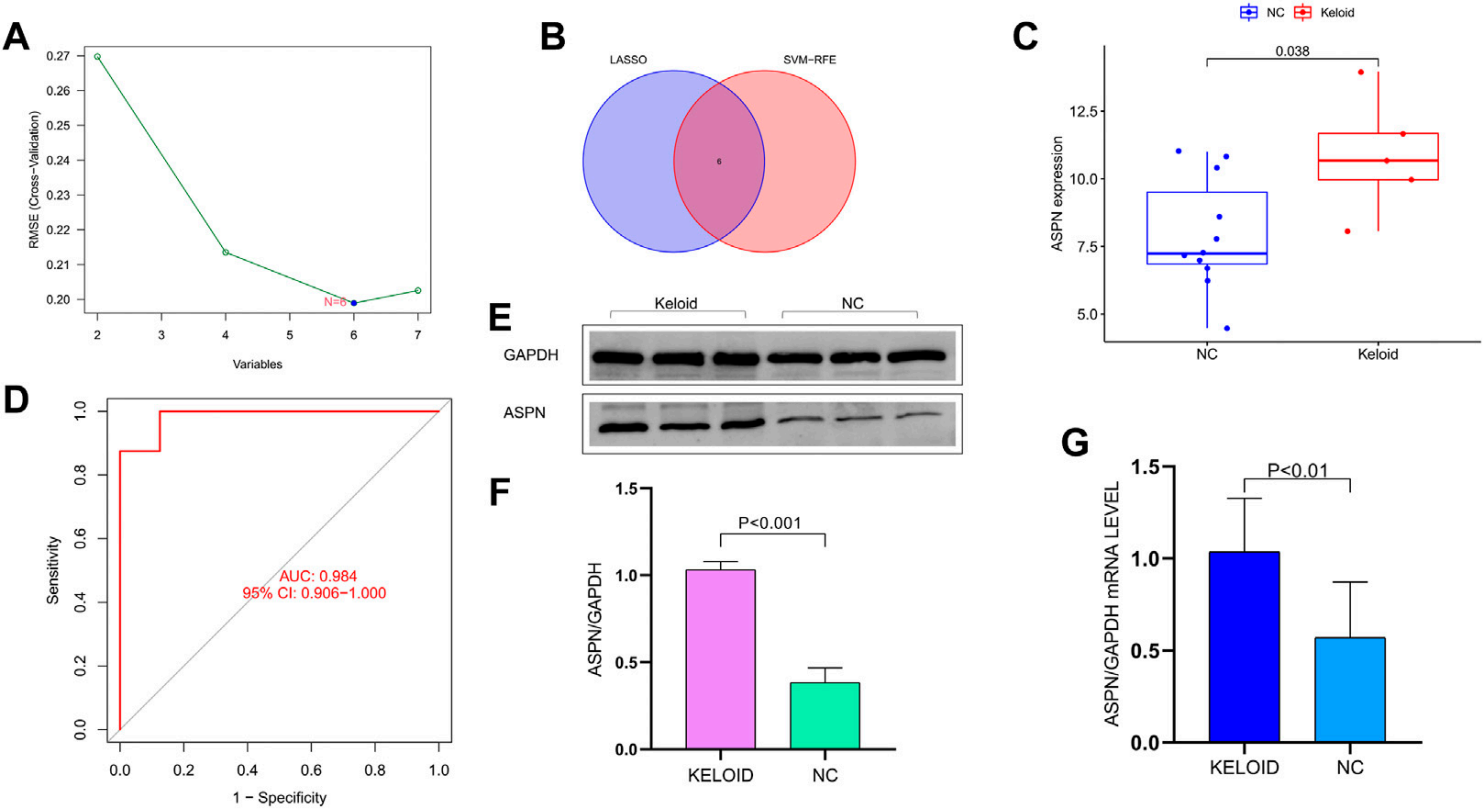
**(A)** The SVM dimensionality reduction algorithm. **(B)** The Venn diagrams shows the six specific genes. **(C)** The boxplot showed significantly higher expression of ASPN in the keloid group than in normal skin. **(D)** The area under the ROC curve is 0.984 in analyzing the sensitivity and specificity of ASPN gene. **(E–G)** Specific expression of ASPN in keloids was confirmed by RT-qPCR and Western blot analysis, ASPN was strongly expressed in keloids but the expression signal was negative or weakly positive in normal skin tissue.

Combining Lasso Cox regression (Figure 3F), SVM dimensionality reduction algorithm (Figure 4A) and Venn diagrams (Figure 4B), six specific genes were shown, which are respectively ASPN, MEST, F2RL2, ACTC1, KRT19, and IL7. The expression of ASPN gene in keloid group and normal control group were analyzed. The boxplot showed significantly higher expression of ASPN in the keloid group than in normal skin.In analyzing the sensitivity and specificity of ASPN gene, the area under the ROC curve was 0.984. Therefore, ASPN was effective in the detection of keloid (Figures 4C,D). In previous studies, there is a very important connection between ASPN and fibrosis, but there are few studies between keloid and ASPN. Therefore, for the above reasons, we opted for ASPN as the target gene (Huang S et al., 2022; Liu L et al., 2021; Tanaka, 2022). The specific expression of ASPN was confirmed in keloid by RT-qPCR, immunohistochemistry and Western blot analysis; ASPN was strongly expressed in keloids, but the expression signal was negative or weakly positive in normal skin (Figures 4E–G). This was also observed in other studies that examined protein or mRNA levels in keloids (Ong CT et al., 2010; Shih B et al., 2010b).

### Screening the optimal siRNA

**FIGURE 5:**
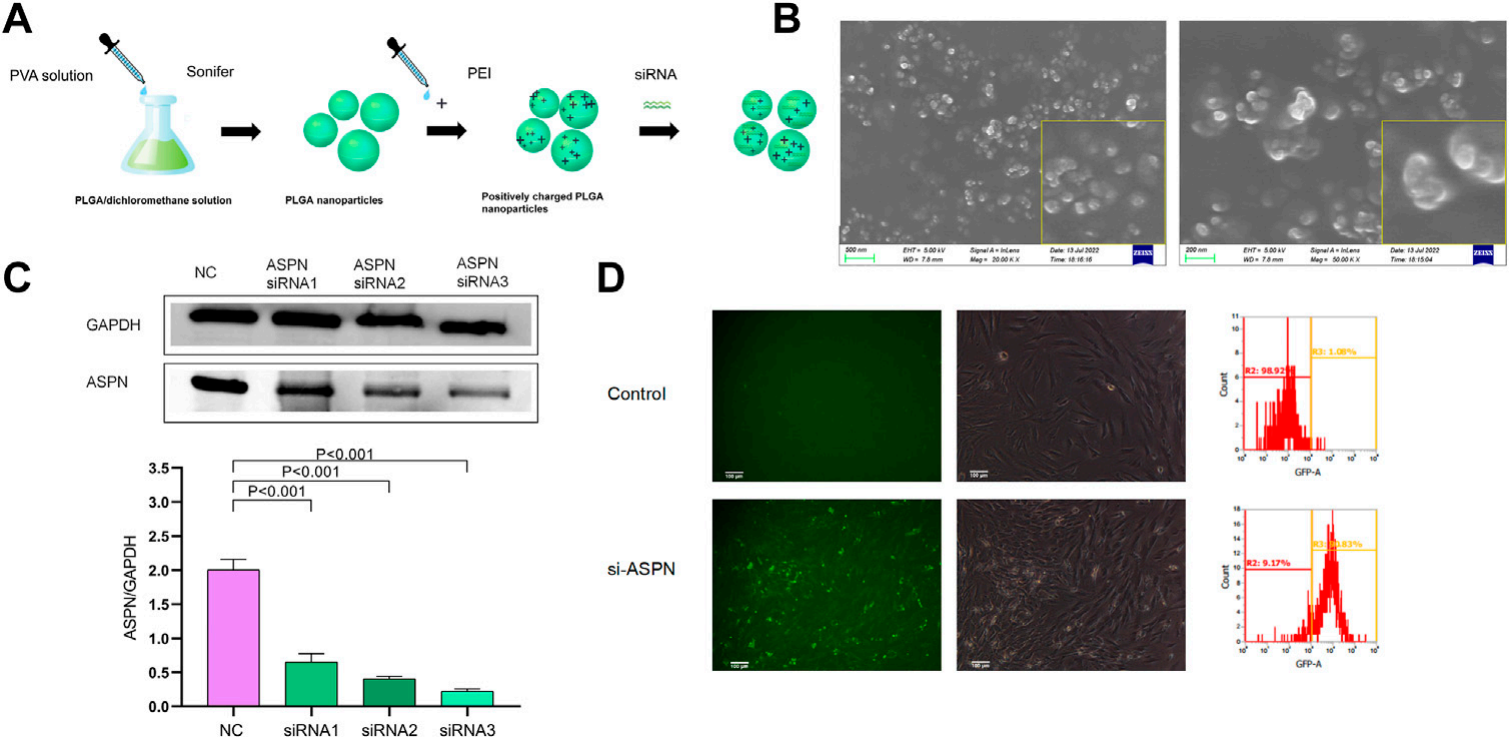
**(A,B)** The PLGA siRNA/nanoparticles under scanning electron microscopy (SEM) were nearly spherical. **(C)** Western blot analysis of keloid fibroblasts transfected with si-ASPN/nanoparticles showed that all three ASPN siRNAs successfully down-regulated ASPN expression and ASPN siRNA3 was the most successful of the three ASPN siRNAs. **(D)** Typical fluorescence and brightfield analyses of ASPN siRNA/nanoparticle (si-ASPN) transfected or untransfected cells were performed and the transfection effectiveness of the ASPN siRNA/nanoparticle complexes was evaluated using flow cytometry. si-ASPN Group showed fluorescence with strong expression capacity.

The PLGA siRNA/nanoparticles under scanning electron microscopy (SEM) were nearly spherical in all cases (Figures 5A,B). Western blot analysis was performed to verify whether keloid fibroblasts transfected with si-ASPN/nanoparticles or not expressed ASPN protein. The expression of ASPN was successfully downregulated by all three ASPN siRNAs. The three ASPN siRNAs inhibited 33.9% (p0.01), 51.6% (p0.01), and 64.9% (p0.05) of the protein level in comparison to the negative control group, respectively (Figure 5C). As a result, ASPN siRNA3 was the most successful of the three ASPN siRNAs. As a result, for further trials, we chose ASPN siRNA3.

#### Transfection efficiency *in vitro*

In Figure 5D, cells tagged with ASPN siRNA/nanoparticles (si-ASPN) or untransfected treatment (Control) were depicted in typical fluorescence and bright field views (Figure 5D). The transfection effectiveness of the ASPN siRNA/nanoparticle complexes was evaluated using flow cytometry. The si-ASPN group displayed fluorescence with a strong expression. These outcomes demonstrated the viability of transfecting keloid fibroblasts with ASPN siRNA/nanoparticle complexes.

#### Cell viability

**FIGURE 6:**
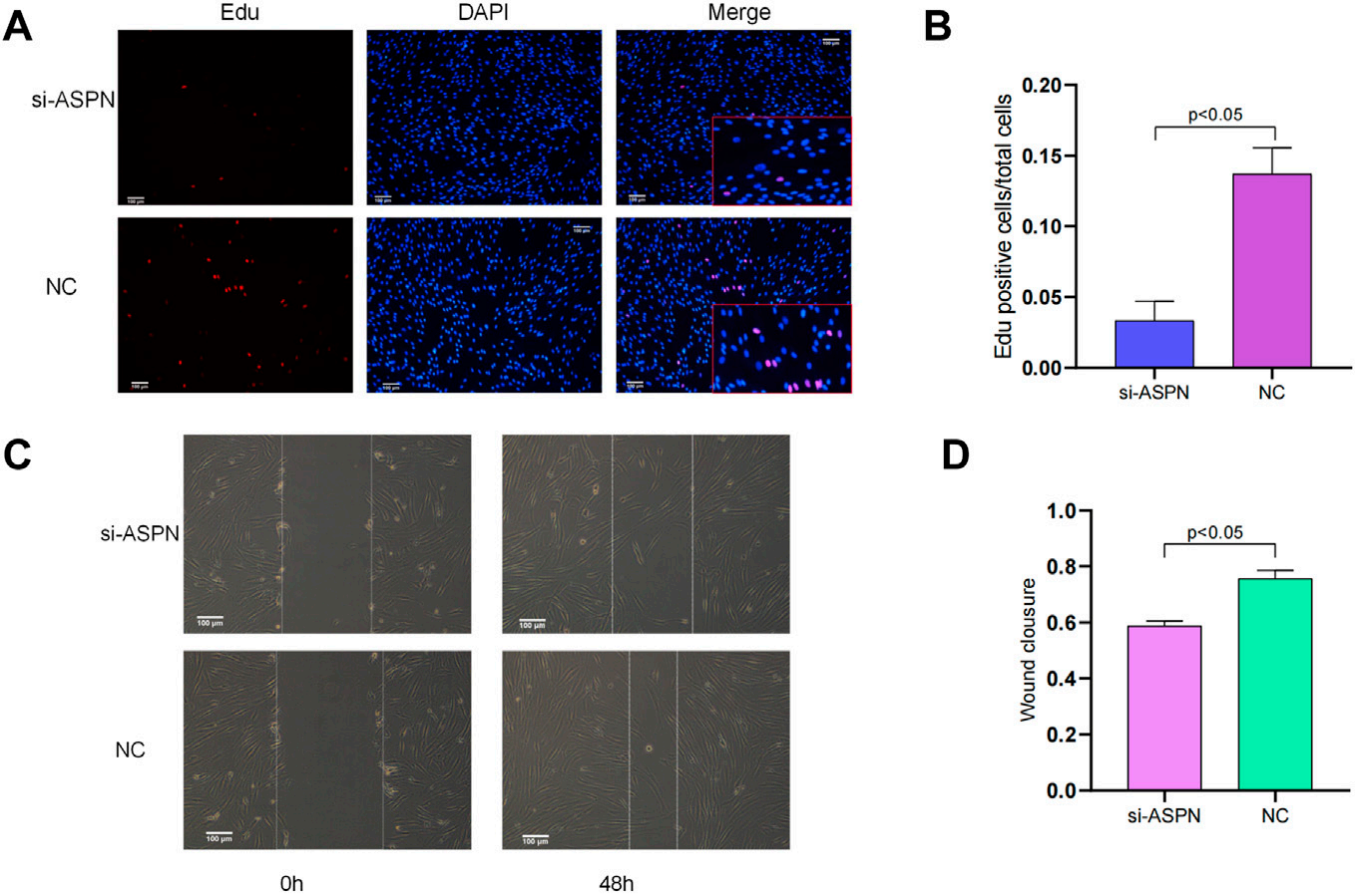
**(A,B)** EdU staining was used to evaluate the proliferation of keloid fibroblasts. The percentage of EdU-positive cells in the si-ASPN group was significantly lower than in the NC group (*p* < 0.05) and the results showed that modulation of ASPN inhibited the proliferation of keloid fibroblasts. **(C,D)** The results of the scratch assay assessing the migration ability of keloid fibroblasts showed that silencing ASPN inhibited the migration of keloid fibroblasts.

EdU staining was used to assess the proliferation of keloid fibroblasts. The percentage of EdU-positive cells in the si-ASPN group was significantly lower than that in the NC group (*p* < 0.05). The ratio of EdU-positive cells were 13.7% ± 1.8% and 3.35% ± 1.36% in the si-ASPN group and NC group respectively. The EdU assay results showed that modulation of ASPN inhibited the proliferation of keloid fibroblasts (Figures 6A,B).

#### Cell migration ability

The migration ability of keloid fibroblasts was evaluated by scratch assay (Figure 6C). After forty-eight (48) h of scratching, the wound healing rate of the keloid fibroblasts treated with si-ASPN (58.76% ± 1.77%) was markedly lower than that of cells in NC group (75.60% ± 2.99%) (*p* < 0.05) (Figure 6D). The results of the scratch assay indicated that silencing ASPN inhibited the migration of keloid fibroblasts.

### *In vivo* effects of the delivery of ASPN-siRNA/nanoparticle complexes

**FIGURE 7:**
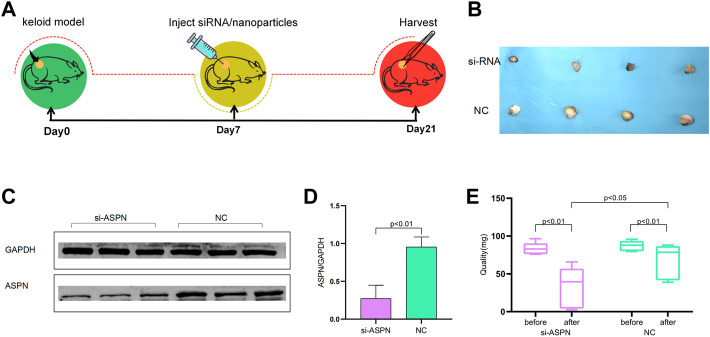
**(A)** Flow chart for *in vivo* experiments. **(B)** External morphological changes of the keloid before and after transplantation. **(C,D)** Western blot examined the expression of ASPN protein in tissues of the si-ASPN and NC groups and revealed that ASPN expression was significantly downregulated after 2 weeks of treatment (*p* < 0.01). **(E)** Quantification of the external morphological changes of the keloid before and after transplantation.

**FIGURE 8:**
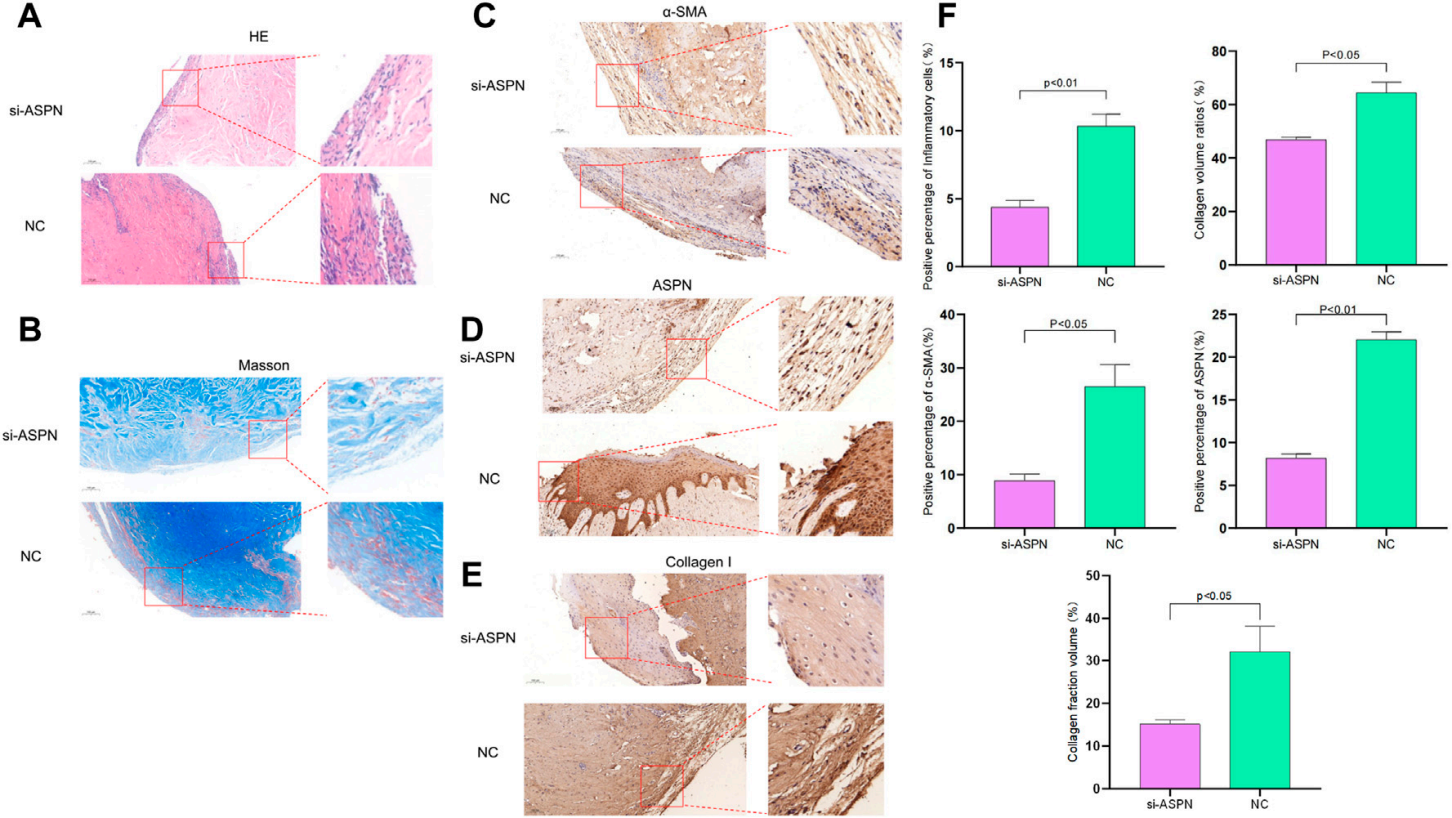
**(A,B,F)** Hematoxylin and eosin (HE) staining and Masson staining showed that collagen cells and fibroblasts in the si-ASPN group were more regular, slender, parallel and evenly spaced compared to the NC group. **(C–F)** Immunohistochemical analysis of ASPN, α-SMA and collagen I was performed in the si-ASPN and NC groups, respectively. Compared to NC group, tissues in the si-ASPN group were negative or weakly positive for ASPN expression and the use of the ASPN siRNA/nanoparticle complexes was effective in reducing fibroblast levels.

External morphological changes of the keloid before and after transplantation was observed and quantified (Figures 7B,E). When keloid tissues from keloid patients were separated into almost identical portions and weighed prior to surgery, it was discovered that the si-ASPN group had an initial mass of 83.82 mg while the NC group had an initial mass of 87.37 mg. The mice were executed 2 weeks after injection, the grafts were removed and weighed again. The weight of the grafts in the si-ASPN group was 34.32 ± 25.90 mg, a reduction of 49.50 ± 23.46 mg. The weight of the grafts in the NC group was 68.75 ± 22.64 mg, a reduction of 18.62 ± 19.01 mg. However, due to the vulnerability of the nude mice, two died in the NC group due to infection and other reasons, so ended up with eight samples from the si-ASPN group and four samples from the NC group.Graft quality decreased both in the si-ASPN and NC groups compared to pre-inoculation quality, but the decrease in graft mass was significantly greater in the si-ASPN group than in the NC group (*p* < 0.01) (Figures 7B,E). To detect the role of ASPN siRNA/nanoparticles, the expression of ASPN protein in tissues was examined from the si-ASPN and NC groups, respectively, using Western blot assays (Figures 7C,D). After isolating and blocking the protein, it was incubated with anti-ASPN antibody. As shown in Figures 7C,D, ASPN expression was significantly downregulated after 2 weeks of treatment (*p* < 0.01). To ascertain the expression of ASPN protein, samples were immune-stained with ASPN antibody 2 weeks after injection of ASPN siRNA/nanoparticles. Tissue sections were then observed under a microscope and images were recorded. The tissues in the si-ASPN group were all negative or weakly positive for ASPN expression signal, whereas the tissues in the NC groups had a more pronounced ASPN expression signal (Figure 8D **)**. Hematoxylin and eosin (HE) staining and Masson staining showed that collagen cells and fibroblasts in the si-ASPN group were more regular, slender, parallel and evenly spaced compared to the NC group (Figures 8A,B **)**. Activated fibroblasts can excrete massive amounts of α-SMA, which promotes the secretion and sedimentation of extracellular matrix (LeeWon et al., 2013; G.Carpino et al., 2005). To a large extent, activated fibroblasts are the exclusive source of collagen I (Michelle Tallquist and Molkentin, 2017; Rana et al., 2020; Qiyao Li et al., 2016). Therefore, 2 weeks after injection, immunohistochemical analysis of α- SMA and collagen I were performed to investigate the effect of ASPN-siRNA/nanoparticle for further study (Figures 8C,E). By contrast, the use of the ASPN siRNA/nanoparticle complexes was effective in reducing fibroblast levels (Figure 8F). These results generally showed that silencing of ASPN resulted in modest antiproliferative of fibroblasts and promoting the more orderly arrangement of collagen fibers.

## Discussion

In this study, the transcriptome sequencing results of keloid and normal skin were compared. Bioinformatics analysis was used to screen a total of 64 DEGs, of which 24 showed up-regulation and 40 showed down-regulation, and the “Wnt signaling pathway” was obtained, among others. Combining Lasso Cox regression, SVM dimensionality reduction algorithm and Venn diagrams, six specific genes were screened out.We then selected ASPN, one of the six genes, for a follow-up study. Research proves that keloid tissues had considerably higher levels of ASPN protein expression and that the Wnt signaling pathway maybe play an important role in keloid development. This suggests that the ASPN gene may control the developmental rate of keloids by modulating the Wnt signaling pathway.

There has been a very important association between ASPN and fibrosis in previous studies, but the relationship between keloids and ASPN has been sparsely studied (Huang S et al., 2022; Liu L et al., 2021; Tanaka, 2022). Due to the rapid development of mechanical biology and the research on the formation and progress of keloids promoted by mechanical forces, the ECM molecular ASPN can also become an effective therapeutic target (Huang C et al., 2012a; Huang C et al., 2012b; Huang C et al., 2013; Deng Z et al., 2021; Syed F et al., 2012). Keloid are also strongly associated with fibroplasia (Huang C et al., 2014; Huang and Ogawa, 2020; Jonathan et al., 2016; DorothySupp et al., 2012). Based on these conclusions, we constructed siRNA/nanoparticle complexes containing ASPN siRNA to investigate the effects of ASPN gene regulation on the keloid fibroblasts. In fibroproliferative diseases, fibroblasts are responsible for increased ECM stiffness (Nelesen et al., 2019). α-SMA is a well-known signature protein used to assess activated fibroblasts in a variety of tissues and organs (LeeWon et al., 2013; G. Carpino et al., 2005). Collagen is the most plentiful structural protein in the human body. While type I collagen (COLI) is the predominant collagen type in connective tissue (Li W et al., 2021). The expression of COLI was significantly higher in keloids compared to the normal group. Therefore, COLI can precisely reflect the growth rate and status of keloid fibroblasts, and its down-regulation can suppress the proliferation of keloid fibroblasts. According to our findings, the si-ASPN group’s -SMA and COLI levels were significantly lower than those of the NC group. Additionally, the EdU assay demonstrated that inhibiting ASPN caused keloid fibroblasts to stop proliferating. This demonstrates that keloid fibroblast proliferation and metabolic activity can be inhibited by a reduction in ASPN protein expression.

Our study developed a xenograft mouse model, and after 14 days, it was discovered that the expression level of ASPN protein in the si-ASPN group had been significantly lower than in the NC group. In comparison to the NC group, the collagen in the si-ASPN group was more homogeneous, slender, parallel, and regularly arranged. This shows that si-ASPN can be delivered from the nanoparticles into the keloid grafts continuously and steadily. The si-ASPN/nanoparticle complexes may therefore be a useful kind of gene therapy for the treatment of keloid. Our investigations’ uniqueness is the utilization of nanoparticles as vector or vehicle to transport si-ASPN. Numerous studies are being done on nanoparticles as non-viral gene delivery vehicles because of their biocompatibility and better regulatory release. Because PLGA nanoparticles can deliver their contents for a long time, they could be used to release genes into keloid fibroblasts (Delgado D et al., 2012; Wong SP et al., 2012; XingGuo et al., 2020). Thus, si-ASPN can be slowly released into keloid tissues and exert therapeutic effects. In fact, both the NC and si-ASPN groups had a decline in graft quality after transplantation, although this decline in graft quality was noticeably larger in the si-ASPN group than in the NC group, according to our findings. We hypothesize that the aforementioned may be related to the immunological health and dietary needs of nude mice. According to the outcomes of our investigation, xenografts were inhibited in the same way by both groups of age-matched nude mice, while the si-ASPN group had a greater decrease in xenograft quality, indicating that si-ASPN has a certain effect. Additionally, earlier research has demonstrated that the ASPN gene is present in both humans and animals, and that si-ASPN functions in xenografts made from nude mice. As a result, we speculate that si-ASPN may have some sort of function in human keloid tissue (Maccarana M et al., 2017).

There are, of course, several limitations to this experiment. One of these limitations is that nude mice only exhibit T lymphocyte immunodeficiency, B cells and NK cells are still functional, and their immune systems are still capable of blocking the growth of external tumors (Wu et al., 2017). Furthermore, for xenografts implanted subcutaneously in nude mice, the blood supply, lymphatic reflux, and delivery of numerous nutrients necessary for xenograft growth are insufficient, which has an impact on the growth of the transplanted xenograft. Moreover, keloid is a progressive proliferative disease, which recurs repeatedly and has a long course of disease. Inadequately, due to the limitations of this experiment, the long proliferation cycle of tissues was not fully expressed in the nude mice experiment. Thus, we assumed that the proliferation cycle of nude mice tissues is very long. Second, we only investigated the effect of ASPN proteins on the regulation of keloid. Whereas keloid genesis may be the result of a combination of factors (Macarak et al., 2021; Jennings et al., 2022; Kumar and Kamalasanan, 2021). Also, as keloids may be linked to genetic factors (Shih and Bayat, 2010a; Glass, 2017) and our numbers are small, it is not possible to strongly demonstrated the significance of ASPN. Based on the results of the trial outside of our animal experiments, further experimental validation remains to be done on whether the results are valid for humans.

Besides, some studies have shown that the Wnt signaling pathway is thought to be the main cause of fibrosis in different organs (Bowley et al., 2007). Therefore, the Wnt signaling pathway may play an important role in keloid. In the upcoming experiments, the role of the Wnt signaling pathway in keloid may be another novel direction of research.

## Conclusion

In this study, keloid fibroblasts and tissues were transfected with ASPN-siRNA to decrease the expression of ASPN protein. The results of the study showed that the growth and metabolic activity of keloid fibroblasts were declined significantly. This suggests that ASPN siRNA/nanoparticles may provide a novel therapeutic strategy for the management of keloid.

## Data Availability

The Genetic/RNA sequencing data for this article were obtained from the NCBI-Gene Expression Omnibus (GEO) database (https://www.ncbi.nlm.nih.gov/geo/).
